# Radial Growth of Trees Rather Than Shrubs in Boreal Forests Is Inhibited by Drought

**DOI:** 10.3389/fpls.2022.912916

**Published:** 2022-06-02

**Authors:** Jingwen Yang, Qiuliang Zhang, Wenqi Song, Xu Zhang, Xiaochun Wang

**Affiliations:** ^1^School of Life, Qufu Normal University, Qufu, China; ^2^College of Forestry, Inner Mongolia Agricultural University, Hohhot, China; ^3^Center for Ecological Research and Key Laboratory of Sustainable Forest Ecosystem Management–Ministry of Education, College of Forestry, Northeast Forestry University, Harbin, China; ^4^College of Forestry, Northwest A&F University, Yangling, China

**Keywords:** climate change, drought, resistance, tree rings, shrub

## Abstract

Of all forest biomes, boreal forests are experiencing the most significant warming. Drought caused by warming has a dramatic impact on species in boreal forests. However, little is known about whether the growth of trees and shrubs in boreal forests responds consistently to warming and drought. We obtained the tree-ring width data of 308 trees (*Larix gmelinii* and *Pinus sylvestris* var. *mongolica*) and 133 shrubs (*Pinus pumila*) from 26 sites in northeastern China. According to the climate data from 1950 to 2014, we determined three extreme drought years (1954, 1967, and 2008). The response difference of radial growth of trees and shrubs in boreal forests to drought was compared using resilience index, moving correlation and response analysis. The results showed that high temperature (mean and maximum temperature) in previous and current growing seasons promoted the growth of *P. pumila*, but inhibited the growth of trees. On the contrary, wetter conditions (higher PDSI) promoted tree growth but were not conducive to *P. pumila* growth in high latitudes. Moving correlation analysis showed similar results. In addition, water deficit was more likely to inhibit *P. pumila* growth in low latitudes. The drought resistance of *P. pumila* was stronger than that of *L. gmelinii* and *P. sylvestris* var. *mongolica*. Therefore, the growth loss and recovery time of *P. pumila* during drought was less than those of trees. We concluded that *L. gmelinii* and *P. sylvestris* var. *mongolica* are more prone to growth decline than *P. pumila* after the drought caused by climate warming. In the future climate warming, shrub growth may benefit more than trees. Our findings are of great significance in predicting the future changes in ecosystem composition and species distribution dynamics in extreme climate susceptible areas.

## Introduction

According to the latest assessment report of IPCC, due to the increase in atmospheric greenhouse gas concentration caused by human activities, the global average surface temperature has increased by 1.09°C by the early 21st century (IPCC, 2021). Climate warming has led to an increasing frequency, amplitude and duration of extreme climate events, which is expected to continue in the future (Li et al, 2020). This poses a key challenge to the stability of forest ecosystems and the multiple ecosystem services they provide (Seidl et al., 2017; Bottero et al., 2021). Extreme climate (such as extreme drought) can affect plant function, primary and secondary growth, tree recruitment and mortality, carbon and water balance of forest ecosystems (Senf et al., 2020; Bottero et al., 2021). Over the past few decades, a decline in global tree growth and an increase in forest mortality has been observed in various forest biomes through severe drought events related to global warming (Choat et al., 2018; Arellano et al., 2019; Senf et al., 2020).

Boreal forests are widespread, accounting for approximately one-third of the global forest area (Sullivan et al., 2021) and play a key role in the global carbon cycle, water cycle and climate change (Gauthier et al., 2015). Especially they are experiencing the most significant warming of any forest biome (IPCC, 2021). Due to significant climate change, the growth of trees in boreal forests has been seriously affected, especially coniferous trees (Mayor et al., 2017; Lopez et al., 2021; Sturm et al., 2022). Many studies have been conducted on the impacts of drought on forests in North America and Europe, but few studies have assessed the impact of drought on boreal forests in Asia (Bottero et al., 2021; Sturm et al., 2022). In addition, there is a significant drought legacy effect on tree growth. In 1–4 years after severe drought events, the recovery time of tree growth can be used to understand the impact of drought on boreal forest ecosystem, including the cycle of carbon feedback on climate change (Anderegg et al., 2013; Camarero et al., 2021a; Iqbal et al., 2021). In some cases, the lag response of tree radial growth to precipitation and temperature anomalies may last for 36–57 months, and high antecedent precipitation improves tree radial growth (Ogle et al., 2015; Jiang et al., 2019). The legacy effect of drought on growth is highly variable, either slowly decreasing or rapidly increasing (Kannenberg et al., 2020). The sensitivity of radial growth to climate change is often manifested in changes in ring width and is closely related to the resilience of drought events (Lloret et al., 2011; Mérian and Lebourgeois, 2011; Duan et al., 2022). The growth resilience of different species varies greatly, depending on the specific physiological response of species to drought events (Anderegg et al., 2013; Gazol et al., 2017, 2018; Duan et al., 2022). However, the resistance and resilience of forest species depend not only on species-specific patterns but also on life forms. Wu et al. (2018) found that the legacy effect of drought on vegetation growth is different for trees, shrubs and grass. Deep-rooted trees showed drought legacy reaction and reduced growth within 4 years after extreme drought. In contrast, the drought legacy effects of shrubs and grasses are about 2 and 1 year, respectively. Species mortality caused by drought is related to species-specific water deficit sensitivity and may lead to changes in species composition, making more drought-tolerant species dominant (Férriz et al., 2021). Therefore, as extreme climate events increase, exploring the response of different species to extreme climate events is crucial to understanding forest dynamics (Babst et al., 2019; Bastos et al., 2020; McDowell et al., 2020; Zweifel et al., 2021).

Trees and shrubs respond differently to global climate change in many natural environments due to their different life forms (Morales et al., 2012; Götmark et al., 2016; Shetti, 2018). Previous studies have shown that shrubs have been more sensitive to climate warming than trees over the past few decades (Morales et al., 2012; Pellizzari et al., 2017). Drought will increase the risk of xylem cavitation of larch and reduce tree vitality (Oberhuber et al., 2014). Therefore, the occurrence of drought is beneficial to pine forests and exacerbates the decline of larch forests in the same area (Dulamsuren et al., 2009). Under the continuous influence of climate warming, pine will gradually replace the natural population of larch in Siberian forests (Urban et al., 2019). Boreal forests are particularly vulnerable to climate change because they are usually located on permafrost (Helbig et al., 2016; Zhang et al., 2019a,b). Meanwhile, tall shrubs are encroaching into alpine and Arctic tundra landscapes, possibly responding to rising air temperatures (Tape et al., 2006; Forbes et al., 2010; Hallinger et al., 2010; IPCC, 2021). Pellizzari et al. (2017) found that the decline in tree growth in the Mediterranean region after the 1980s may be caused by drought stress exacerbated by climate warming, but the drought will not affect juniper trees. We also found that larch and pine trees are more vulnerable to global warming than shrubs (Yang et al., 2020). The negative effect of temperature in growing season on the growth of larch and pine is greater than that on *Pinus pumila*, and the promotion effect of precipitation in winter and spring on *P. pumila* is the greatest (Yang et al., 2020). However, it is not entirely clear whether the trees and shrubs in boreal forests of China have such different responses to extreme climate change.

*Pinus sylvestris* var. *mongolica* and *Larix gmelinii* are important tree species in boreal forests of Asia (Zhang et al., 2019a), and *P. pumila* is an important shrub species in boreal forests. In recent decades, drought has occurred frequently in northeast China, which strongly impacts on the radial growth of major tree species in boreal forests, especially in semi-arid sites (Zhang et al., 2021). After a serious drought, water-sensitive trees usually reduce their radial growth and recover previous growth when subsequent rainfall increases (Fang et al., 2017). To clarity, the impact of drought events on the growth of different species and life-form species in boreal forests. We investigated the growth pattern of three main species in boreal forest, northeast China: Dahurian larch (*L. gmelinii*), Mongolian pine (*P. sylvestris* var. *mongolica*), Siberian dwarf pine (*P. pumila*) and their response to climate change. The study aims to compare the response of tree and shrub growth to drought to help predict the future dynamics of boreal forests and feedback to the global climate system. We hypothesize that: (1) the main climate limiting factors of radial growth of *L. gmelinii*, *P. sylvestris* var. *mongolica* and *P. pumila* are different; (2) Compared with shrubs, tree growth has strong resistance to drought and weak recovery and resilience; (3) After a drought, the recovery time and total growth reduction in radial growth of tree species are less than those of shrubs.

## Materials and Methods

### Filed Sampling

**Figure 1:**
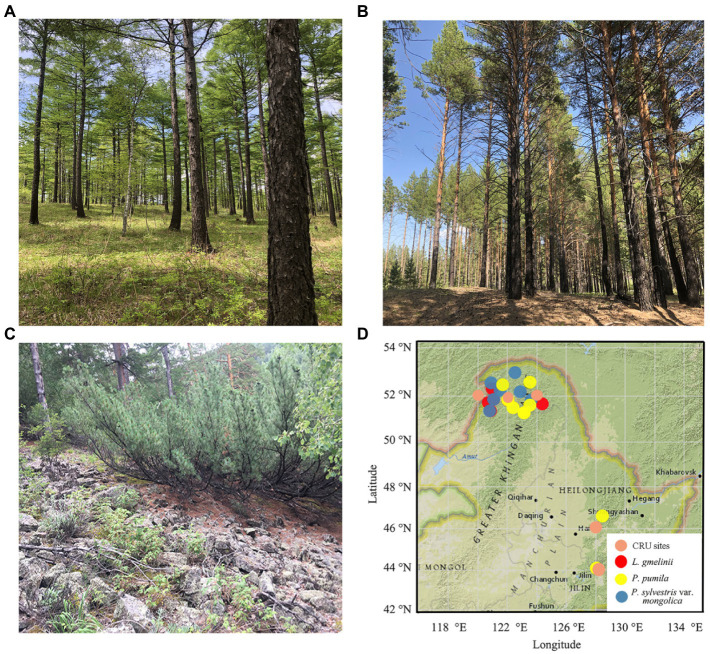
Field photos (**A**: *Larix gmelinii*; **B**: *Pinus sylvestris* var. *Mongolica*; **C**: *Pinus pumila*) and location map of tree–ring sampling sites **(D)** (red circle, *Larix gmelinii*; yellow circle, *Pinus pumila*; blue circle, *Pinus sylvestris* var. *mongolica*) in the northeast of China. Fieldwork was conducted from 2015 to 2018 at 26 sites.

The study area is located in the boreal forests of northeastern China, with a latitude range from 44°06′ to 52°59′ N and a longitude from 120°44′ to 128°28′ E (Table 1, Figure 1). It belongs to the cold temperate continental monsoon climate, and it is cool and rainy in summer and cold and dry in winter. The mean annual precipitation (1950–2014) is between 415.6 and 636.2 mm, and more than 68% occurs from June to August. The mean annual temperature ranges from −6.3°C to 0.5°C. January is the coldest month (mean minimum temperature −38.2°C at ZL site), and July is the warmest month (mean maximum temperature 25.3°C at TS site). The annual frost-free period is 80–120 days, with early and late frost in September and May.

**Table 1:** Information on the 26 sample sites in the northeast of China.

Site	Species	Code	Latitude (N)	Longitude (E)	Altitude (m)	Sample number (tree/core)
Xinlin	*Larix gmelinii*	XLLG	51°40′	124°23′	513	20/33
Yikesama		YKLG	51°51′	121°05′	823	20/35
Mo’erdaoga		MELG	51°22′	120°52′	1,240	18/31
Qiqian		QQLG	52°19′	121°00′	578	23/41
A’long Mountain		ALLG	51°50′	122°03′	820	18/30
Angelin		AGLG	51°44′	120°44′	747	27/54
Yikesama	*Pinus sylvestris* var. *mongolica*	YKPS	51°51′	121°05	823	25/45
Mo’erdaoga		MEPS	51°22′	120°49′	1,072	25/49
Qiqian		QQPS	52°34′	120°54′	535	25/47
Yong’an Mountain		YAPS	52°14′	121°30′	597	24/45
Feihu Mountain		FHPS	52°11′	122°52′	790	24/48
Fuke Mountain		FKPS	52°28′	121°40′	626	21/42
Mohe		MHPS	52°59′	122°32′	435	16/32
Shilin		SLPS	51°50′	123°37′	913	22/44
Laobai Mountain	*Pinus pumila*	LBHPP	44°06′	128°03′	1,685	12/36
Laobai Mountain		LBLPP	44°06′	128°03′	1,531	12/34
Tao Mountain		TSPP	46°38′	128°28′	1,369	15/42
Xiaobai Mountain		XBPP	51°37′	123°32′	1,400	15/58
Hanma		HMPP	51°31′	122°24′	900	18/72
Fuke Mountain		FKPP	52°28′	121°40′	1,096	10/32
A’long Mountain		ALPP	51°50′	122°03′	1,104	7/28
A’long Mountain		AHPP	51°51′	122°03′	1,506	7/28
Zhalinku’er		ZLPP	52°36′	123°33′	941	7/28
Dabai Mountain		DLPP	51°18′	123°09′	1,362	10/34
Dabai Mountain		DMPP	51°18′	123°08′	1,431	10/33
Dabai Mountain		DHPP	51°18′	123°08′	1,530	10/24

The study area is mainly the boreal forest dominated by *L. gmelinii* and accompanied by *P. sylvestris* var. *mongolica*, *Betula platyphylla*, and *Populus davidiana*. Shrubs mainly include *P. pumila*, *Rhododendron dauricum*, *Vaccinium vitis-idaea*, *Rosa dahurica*, et al. The main herbs include *Maianthemum bifolium*, *Sanguisorba officinalis*, and *Trientalis europaea*, et al. The soil is brown coniferous forest soil. *Pinus sylvestris* var. *mongolica* and *L. gmelinii* are coniferous trees, and *P. pumila* is a creeping shrub with many trunks. *Pinus pumila* mainly grows in two vegetation types: they are high-altitude subalpine plants and low-altitude canopy trees. The former grows in the subalpine zone with fruticulose and herbaceous plants under its canopy. The latter grows under the canopy of arboreal trees (e.g., *L. gmelinii*, *P. sylvestris* var. *mongolica*, and *Betula ermanii*; Okuda et al., 2008). The subalpine krummholz forest dominated by *P. pumila* is mainly distributed in the altitude range of 900–1,500 m in the Daxing’an Mountains.

### Tree-Ring Sampling and Chronology Development

At each site, 32–54 tree-ring cores were collected at breast height from healthy trees using a 5.15-mm-diameter increment borer. Two cores were collected from each tree. One disc including the entire stem cross section was collected from the base of *P. pumila* trunks with a handsaw. Discs were obtained only from the largest trunk of isolated, mature, healthy *P. pumila* individuals. A total of 576 cores from 126 *L. gmelinii* and 182 *P. sylvestris* var. *mongolica* and 133 discs from *P. pumila* were sampled at the 26 sites.

To remove the non-climate signals related to age or the effects of stand dynamics, each ring-width series were detrended and standardized by fitting a negative exponential curve or linear line using the ARSTAN program (Cook and Holmes, 1986). The tree-ring index was obtained by dividing the ring width by the fitted value for each ring. All detrended series were averaged to chronologies using the bi-weight robust mean (Cook and Holmes, 1986). The standard chronologies (STD) were used in the subsequent analyses.

Statistical analyses were used to compare chronologies among localities and species for the period 1950–2014. The expressed population signal (EPS), defined as the proportion of each series signal of the total series variance, was used to quantify the reliability of the chronology (Wigley et al., 1984). The mean sensitivity (MS) and the first-order autocorrelation (AC1) were calculated on the detrended individual index series and averaged to measure the year-to-year variability and how current-year growth was influenced by previous-year climatic factors. The mean series correlation between trees (Rbar) allowed us to evaluate the strength of the common growth signal over time. The standard deviation (SD) of inter-annual ring width variability was calculated as a proportion of mean ring-width. Variance in the first eigenvector (VF1) of all series identifies the common growth variability among all trees at each site. The signal-to-noise ratio (SNR) is a measure of the strength of the common high-frequency signal in the ring-width indexes of trees from the same site.

### Climate Data

We used CRU TS 3.23 0.5° × 0.5° gridded monthly and seasonal temperature and precipitation data to analyze growth–climate relationships for the period 1950–2014 because no nearby weather stations exist. The data were extracted from the sample area using the KNMI Climate Explorer web page.^1^ The CRU database is formed by interpolated values from regional meteorological stations. In areas with a low density of weather stations CRU data contained inhomogeneities (McAfee et al., 2014), especially precipitation in alpine regions. Therefore, we verified the CRU precipitation data with the correlation between the observation data of the meteorological station and the CRU precipitation data. The monthly total precipitation (P), mean (*T*_mean_), minimum (*T*_min_), and maximum temperature (*T*_max_) were used to analyze growth-climate relationships. Seasons were defined as: winter is from December of the previous year to February of the current year, spring as March–May, summer as June–August, and autumn as September–November.

### Statistical Analyses

The Pearson correlation was used to determine the relationship between the tree-ring index and monthly and seasonal climate variables to determine the main climate factors that limit the radial growth of each species. Radial growth is affected by the current and previous year’s climate (Fritts, 1976). Therefore, climate variables over 12 months, from November of the previous year to October of the current year, were used for the correlation analysis. To investigate the temporal stability of growth–climate relationship, we carried out a moving 21-year window correlation analysis using DENDROCLIM2002 to analyze the temporal stability of dendroclimatic relations (Biondi and Waikul, 2004).

To analyze the radial growth of *L. gmelinii*, *P. sylvestris* var. *mongolica*, and *P. pumila* responses during and after drought events, resistance, resilience, and the relative resilience were calculated (Lloret et al., 2011). These drought events were selected based on the PDSI (Palmer Drought Severity Index). We used the resistance, recovery and resilience indices defined by Lloret et al. (2011) to quantify individual tree responses to the drought events.

*R*t = Dr/PreDr*Rc* = PostDr/Dr*Rs* = PostDr/PreDr*RRS* = ((PostDr – Dr)/PreDr)

where *PreDr* and *PostDr* indicate the mean ring width before and after three drought years, respectively; *Dr* indicates the ring width in the drought year.

A retrospective study of tree-ring widths allowed us to calculate resistance, resilience and recovery indices for three drought events: 1954, 1967, and 2008. We used the length of the growth recovery time (GRT) and total growth reduction that were put forward by Thurm et al. (2016) and Móricz et al. (2021). GRT represents the time (unit is the year) required to recover the predrought growth level again, including the drought years. TGR means the total growth reduction caused by the drought, and we calculated the TGR index, including drought year and the accumulated loss of growth during GRT.

## Results

### Comparison of the Chronological Characteristics in Different Species

**Figure 2:**
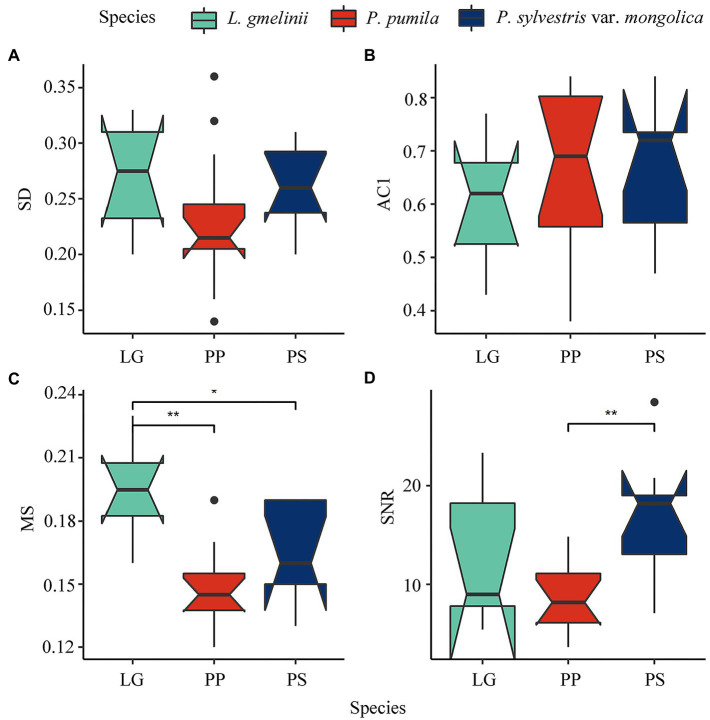
Boxplot of three species chronology main statistics (**A**: SD; **B**: ACI; **C**: MS; **D**: SNR) in this study, ^**^ represent *p* < 0.01; ^*^ represent *p* < 0.01, those without ^*^ markings are not significant. SD, standard deviation; AC1, first order autocorrelation; MS, mean sensitivity; SNR, signal-to-noise ratio.

Sampled *L. gmelinii* and *P. sylvestris* var. *mongolica* were older than *P. pumila*, XLLG had the highest SNR, and SLPS had the highest VF1 and Rbar (Supplementary Table S1). The AC1 of *P. pumila* and *P. sylvestris* var. *mongolica* was higher than that of *L. gmelinii*, while the MS of *L. gmelinii* was the highest among the three species (Figure 2). Statistics related to the common growth signal and the mean correlations among individuals within each site (Rbar, EPS, VF1, SNR, and SD) were usually higher for the trees than the *P. pumila*. All analyses indicated that the 26 chronologies were rich in climatic signals and suitable for analyzing growth–climate relationships.

**Figure 3:**
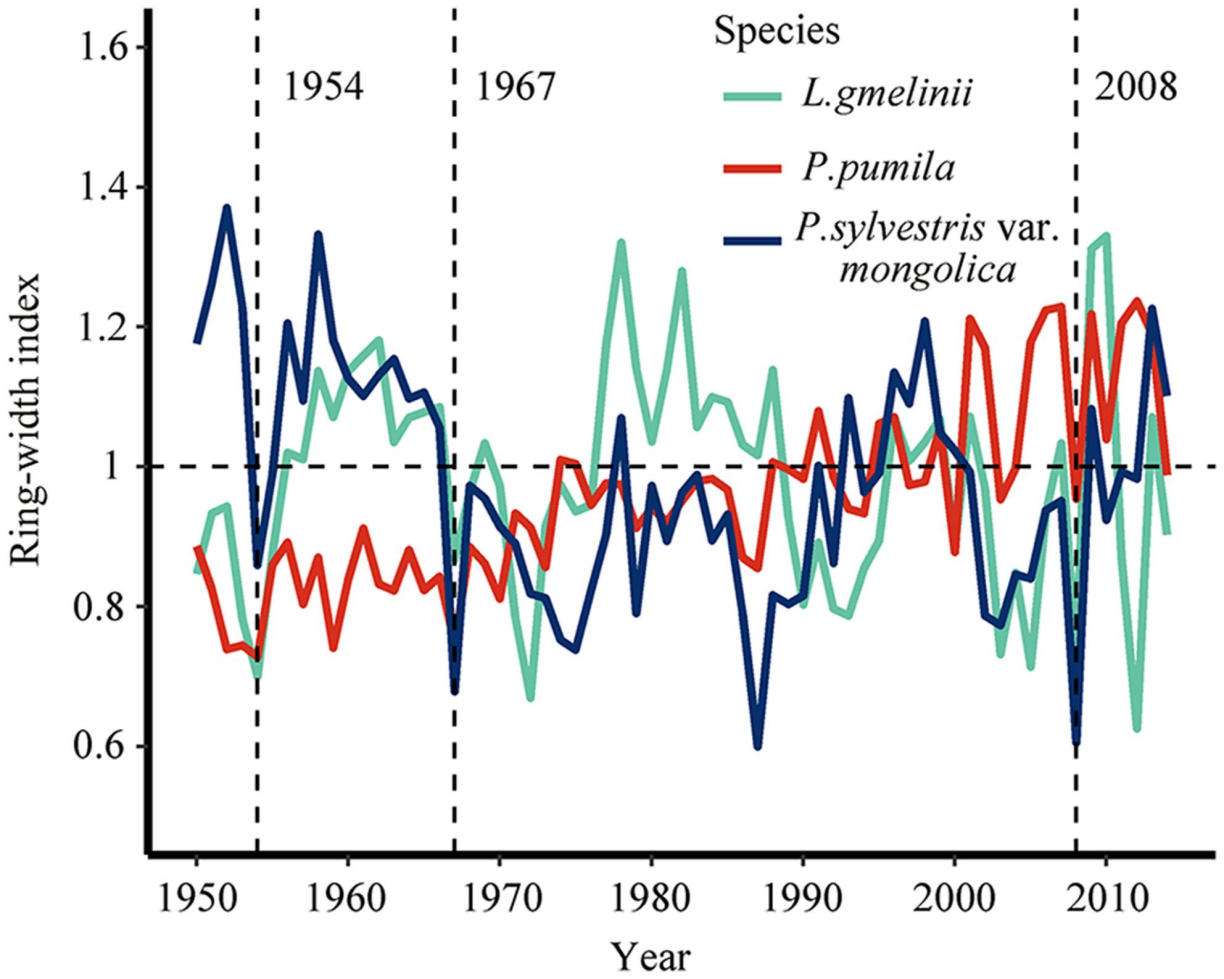
Changes in the ring-width index of *Larix gmelinii* (green line), *Pinus pumila* (red line), and *Pinus sylvestris* var. *mongolica* (blue line) in northeast China, the vertical dash lines represent drought years (1954, 1967, and 2008).

The ring-width chronology of *P. pumila* showed a lower Rbar for growth between individuals than tree species indicating that its radial growth is less consistent than that in *P. sylvestris* var. *mongolica* and *L. gmelinii* (Supplementary Table S1). There is a high degree of consistency between the chronologies of the same species (Supplementary Figure S1). In 1954, 1967, and 2008, the three species formed narrower rings (Figure 3 and Supplementary Figure S2). The chronological consistency of *L. gmelinii* was higher than that of *P. sylvestris* var. *mongolica* and *P. pumila*. The year 1986 was not considered a drought year for all three species because the ring-width index of *L. gmelinii* was greater than 1 (Figure 3).

### Growth–Climate Relationships of Different Species

**Figure 4:**
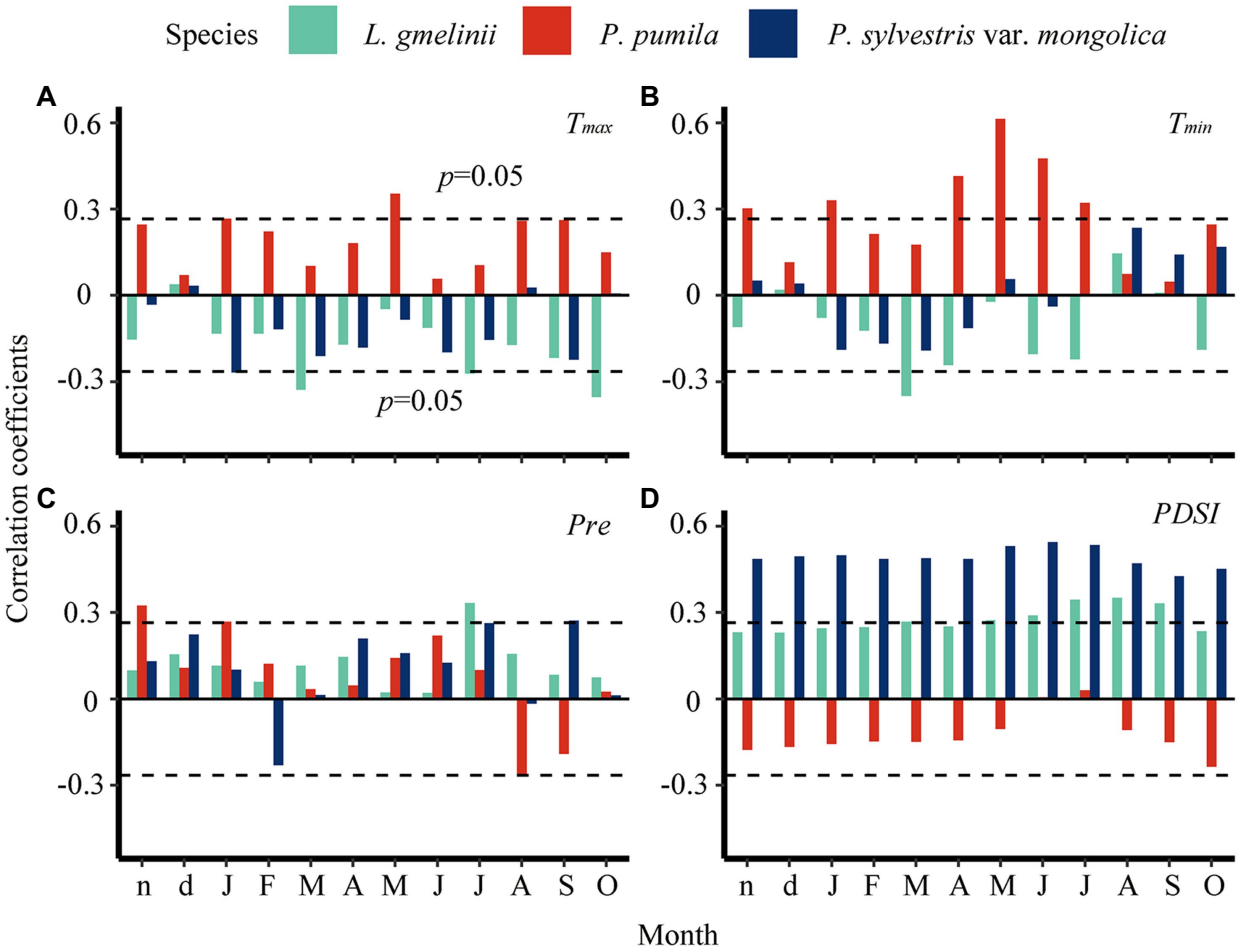
Pearson correlation of ring-width index with monthly climate data from previous November (lower case n) to current October (upper case O) during 1950–2014. Horizontal dashed lines indicate the 95% significance levels. *T*_max_–monthly maximum temperature **(A)**, *T*_min_–monthly minimum temperature **(B)**, *Pre*–monthly precipitation **(C)**, *PDSI*–Palmer Drought Severity Index **(D)**.

The growth of *L. gmelinii* and *P. sylvestris* var. *mongolica* was negatively correlated with maximum temperature in the previous winter (Figure 4A and Supplementary Figures S3, S4). In contrast, high temperatures in the previous winter were related to increasing *P. pumila* ring widths (Supplementary Figures S5, S6). Warm spring and summer conditions were not conducive to the radial growth of *L. gmelinii* and *P. sylvestris* var. *mongolica*. However, *P. pumila* was positively correlated with minimum temperature in the current growing season (Figure 4B and Supplementary Figures S4, S5). Wet conditions in the previous winter enhanced the radial growth of *L. gmelinii* and *P. sylvestris* var. *mongolica* but had an inhibiting effect on the *P. pumila* (Figures 4C,D). The impact of winter precipitation on *P. pumila* radial growth was greater than for larch and pine. In addition, wet conditions (high PDSI) was more beneficial to the radial growth of larch and pine than *P. pumila*, which indicated that trees are more sensitive to moisture (Figure 4D and Supplementary Figures S3–S5).

**Figure 5:**
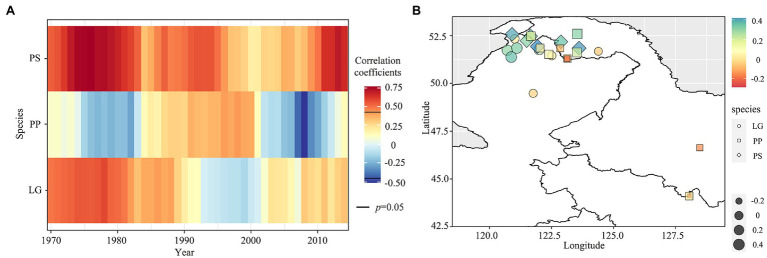
The 21-year moving correlation analysis between the ring-width index of three species and minimum PDSI from June to August during the period 1970–2014 **(A)**; the person correlation between the ring-width index and minimum PDSI from June to August in different sites **(B)**.

Moving 21-year window correlation analysis results indicated that *P. sylvestris* var. *mongolica* was positively correlated with PDSI, with a decreasing correlation between 1995 and 2005 and increasing in the last decade (Figure 5A and Supplementary Figure S7). However, the positive correlation between *L. gmelinii* and PDSI turned negative after 1990 and a positive correlation in the recent decade. Unlike the two arboreal conifers, the correlation between *P. pumila* and PDSI changed from negative to positive around 1980 and shifted to significant negative around 2000. Larch at low latitudes was negatively correlated with PDSI, and *L. gmelinii* and *P. sylvestris* var. *mongolica* at high latitudes were positively correlated with PDSI (Figure 5B). There was a negative correlation between PDSI and *P. pumila* at low latitudes and a positive correlation between PDSI and *P. sylvestris* var. *mongolica* and *L. gmelinii* at high latitudes.

### Resistance, Recovery Time, and Growth Changes of Different Species to Drought Events

**Figure 6:**
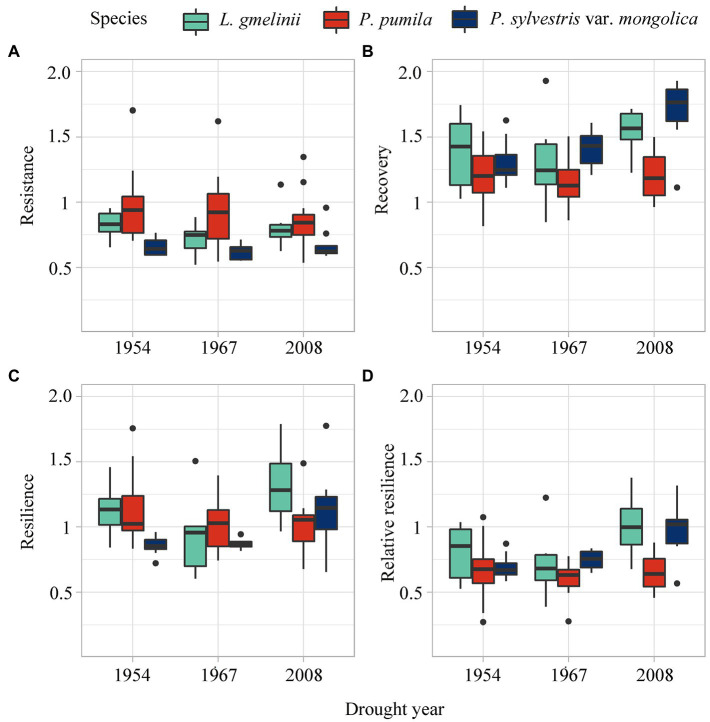
Resistance **(A)**, recovery **(B)**, resilience **(C)**, and the relative resilience **(D)** of *L. gmelinii*, *P. pumila* and *P. sylvestris* var. *mongolica* response to three drought events.

The resistance of *L. gmelinii* and *P. pumila* was stronger than *P. sylvestris* var. *mongolica* (Figure 5), indicating that *L. gmelinii* and *P. pumila* have less growth loss than *P. sylvestris* var. *mongolica* during drought events (Figure 6). The strong recovery of *P. sylvestris* var. *mongolica* suggests that it could recover faster after drought in 1967 and 2008. In 2008, *L. gmelinii* showed the highest resilience, and in 1954, *P. sylvestris* var. *mongolica* showed the lowest resilience. In addition, *L. gmelinii* showed high relative resilience during the three drought events. Along the latitudinal gradient, *P. pumila* resistance increased and resilience decreased with decreasing latitude, while *L. gmelinii* and *P. sylvestris* var. *mongolica* showed the opposite trend (Supplementary Figure S9).

**Figure 7:**
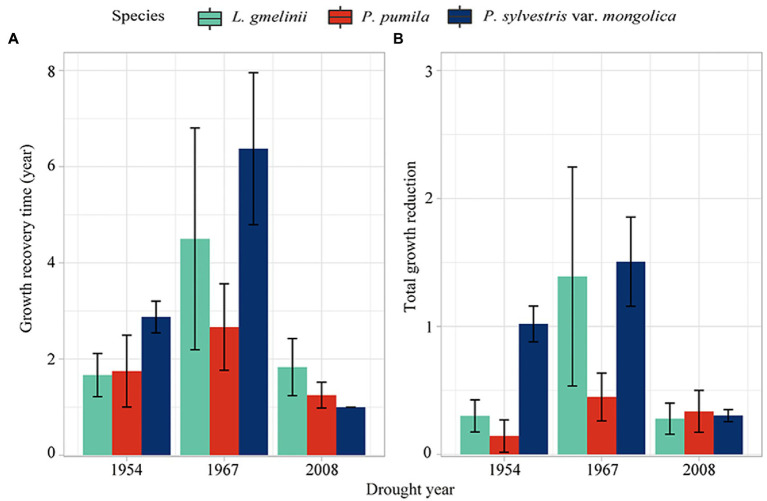
The growth recovery time (GRT) **(A)** and total growth reduction (TGR) **(B)** of *L. gmelinii*, *P. pumila* and *P. sylvestris* var. *mongolica* radial growth to drought events.

In 1954 and 1967, *P. sylvestris* var. *mongolica* needed the longest recovery time; as a result, the *P. sylvestris* var. *mongolica* had the maximum total growth reduction during drought (Figure 7). In 2008, the growing loss of all three species was similar, while the recovery time of *L. gmelinii* was longer. In 1954 and 1967, *P. pumila* had the minimum growth reduction, and the recovery time of *P. pumila* was in the middle of the three drought events.

## Discussion

### Differences in the Responses of Three Species to Climate Change

Among the three species in boreal forests widely distributed in northeast China, the growth of *P. pumila* showed an upward trend, the growth of *P. sylvestris* var. *mongolica* showed a downward trend, and the growth of *L. gmelinii* was relatively stable. *Pinus pumila* is mainly distributed in treeline, while *L. gmelinii* and *P. sylvestris* var. *mongolica* are distributed in relatively lower altitudes. Thus, tree growth could be restricted by water deficits due to climate warming, while shrub growth is promoted by rising temperature (Miller et al., 2017; Harvey et al., 2020). Radial growth is a sensitive and closely tracked clue to climate change (Camarero et al., 2021a). Shrub and tree growth trends reveal differences in the response of different life form species to climate change, which has been reported in polar, alpine, and Mediterranean biological communities (Pellizzari et al., 2017; Treml et al., 2019; Lopez et al., 2021; Šenfeldr et al., 2021). Some studies have found that warming can promote shrub growth and expansion (Hallinger et al., 2010; Frost and Epstein, 2014). Physiological differences between trees and shrubs often lead to different strategies for growth response to climate change (Lyu et al., 2017; Zhirnova et al., 2020; Li et al., 2021). Trees respond more strongly to climate change in the macro-environment, and shrubs are more sensitive to microclimate near the ground because of their dwarfism (Pellizzari et al., 2017; Šenfeldr et al., 2021). During the growing season, trees are exposed to higher air and stem temperatures than shrubs, and the growing season of shrubs is shorter than that of trees (Gazol and Camarero, 2012). Although only one shrub of *P. pumila* was used in this study, it is representative in the timberline of northeast China and can represent the response of other timberline shrubs to some extent. Therefore, the growth of *P. pumila* was positively correlated with temperature, while that of *L. gmelinii* and *P. sylvestris* var. *mongolica* are the opposite. In addition to different phenological periods, the biomass allocation patterns of shrubs and trees are also different (Treml et al., 2019). The proportion of photosynthetic tissue in the stems of shrubs is larger than that of trees (Šenfeldr et al., 2021).

*Larix gmelinii* and *P. sylvestris* var. *mongolica* negatively correlate with spring and summer temperature (Figure 4), and this signal is more obvious in *L. gmelinii*. Precipitation has little effect on the radial growth of *P. pumila.* This is consistent with Šenfeldr et al. (2021)’s results. They found that precipitation is much less important for trees and shrubs growth than temperature. The two tree species have different defoliation patterns and different ways of dealing with climate change, among which *P. sylvestris* var. *mongolica* is more sensitive to water deficits. Defoliation results from plants adapting to seasonal stresses such as drought or low temperature (Sousa-Silva et al., 2018). As a comprehensive effect of temperature and precipitation, both *L. gmelinii* and *P. sylvestris* var. *mongolica* had a significant positive correlation with PDSI. Previous studies have found that *L. gmelinii* and *P. sylvestris* var. *mongolica* have similar growth–climate responses in Siberia (De Grandpré et al., 2011; Belokopytova et al., 2021).

The response of *P. sylvestris* var. *mongolica* growth to temperature rise is closely related to the increased early spring water availability. Drought in winter is a severe threat to the growth of cold-tolerant trees, with boreal trees in winter for up to 5 months annually. Therefore, if there is enough water in the early growing season, trees may be more able to withstand the stress caused by warming and even increase their growth (Zhang et al., 2019a). Because of fallen leaves, the growth of *L. gmelinii* seems to start later than that of *P. sylvestris* var. *mongolica.* Due to the rapid melting of snow in the early growing season, *P. sylvestris* var. *mongolica* benefits more from snowmelt than larch in various environments (Belokopytova et al., 2021). Deciduous traits enable *L. gmelinii* to maintain water in early spring by reducing transpiration loss, while *P. sylvestris* var. *mongolica* with evergreen needles needs sufficient water supply in the early growing season (Rossi et al., 2009; Li et al., 2021). The negative correlation between *P. pumila* growth and PDSI may be due to more precipitations and higher air humidity at higher altitudes.

### Responses of Tree and Shrub Growth to Drought Events

*Pinus pumila* growth is less affected by drought, while trees are more vulnerable to extreme drought (Figure 6). Water stress caused by climate change has triggered a pervasive increase in large-scale tree diebacks and mortality events worldwide (Lewis et al., 2011; Anderegg et al., 2019; Camarero et al., 2021b). Tall trees need an effective long-distance transport channel to resist gravity and friction to transport water from soil to leaves (Choat et al., 2018). In contrast, shrubs require less water because of their low height, shorter paths, and lower soil water potential. In addition, larger tree crowns are more exposed to the canopy positions resulting in higher evaporation and reduced drought tolerance (Kunert et al., 2017; McGregor et al., 2021).

Among the three species, *P. pumila* has the highest resistance and the lowest recovery to drought. Compared with the growth of the two tree species, *L. gmelinii* had higher drought resistance, while *P. sylvestris* var. *mongolica* had higher drought recovery in 1967 and 2008. Zhang et al. (2021) reported that *L. gmelinii* experienced frequent extreme drought had lower drought resistance and higher resilience and was better adapted to extreme droughts. *P. pumila* is distributed at high altitudes and usually grows on steep slopes or shallow rocky soils. Dwarfism makes *P. pumila* drought resistant, but its habitat characteristics make it difficult to recover after drought. The differences in foliage habits (evergreen and deciduous) and strategies to deal with water deficit between *P. sylvestris* var. *mongolica* and *L. gmelinii* resulted in lower drought resistance and greater growth loss *P. sylvestris* var. *mongolica* (Belokopytova et al., 2021). Isohydric *P. sylvestris* immediately regulates transpiration through stomata closure to prevent massive xylem embolism, resulting in reduced photosynthetic rate and growth (Irvine et al., 1998). Conifers are usually more sensitive to stomatal regulation, but larch is less sensitive than pine (Dulamsuren et al., 2009; Khansaritoreh et al., 2018). Anisohydric larch maintains active transpiration to maintain a high photosynthetic rate rather than sacrificing needles and fine roots during severe droughts (Piper and Fajardo, 2014). Therefore, the growth loss of *P. sylvestris* var. *mongolica* was greater than that of *L. gmelinii* in drought events. In addition, besides the drought adaptation of root and stem xylem (Chenlemuge et al., 2013), reducing transpiration through needle abscission and stomatal closure also is an effective drought resistance strategy (Khansaritoreh et al., 2018). Li et al. (2021) found that *L. gmelinii* had a similar drought resistance mechanism. However, most plants die due to high hydraulic damage caused by drought rather than reduced carbohydrate storage (Anderegg et al., 2013; Sperry and Love, 2015; Gessler et al., 2017).

Droughts in the 21st century are likely to become more widespread, intense and persistent (Lévesque et al., 2013; Swenson et al., 2017; Stovall et al., 2019; Ault, 2020). Drought can lead to reduced tree growth and significantly increased mortality (Fang and Zhang, 2019). The recent increase in drought stress seems to have resulted in the transfer of the natural distribution of larch to high latitudes (Mamet et al., 2019). However, for some drought-tolerant shrubs, drought may put them in a favorable position in interspecific competition to replace trees at high altitudes and latitudes (Myers-Smith et al., 2015). Therefore, compared with trees, future warming may be conducive to the growth and distribution of shrubs. In addition, *L. gmelinii* and *P. sylvestris* var. *mongolica* may be replaced by *Larix sibirica* or fast-growing deciduous broadleaf trees in some areas (Kharuk et al., 2009; Mack et al., 2021). Due to drought, the shifts in forest structure may affect the carbon balance and provide positive/negative feedback for warming at the regional level.

## Conclusion

Different species developed different growth strategies to cope with climate change. Water deficit was the dominant limiting factor for the radial growth of *P. sylvestris* var. *mongolica*; however, the effect on *P. pumila* was less. Future warming and drought will likely inhibit the growth of pine and larch. There was a negative correlation between PDSI and *P. pumila* at the low latitude and a positive correlation between PDSI and *P. sylvestris* var. *mongolica* and *L. gmelinii* at high latitude. The resistance of *L. gmelinii* and *P. pumila* was stronger than that of *P. sylvestris* var. *mongolica* and had less growth loss and shorter recovery times during the drought. Comparing differences in the resistance of shrubs and trees to drought events can help us better predict and understand the response of changing ecosystem dynamics to warming.

## Data Availability Statement

The original contributions presented in the study are included in the article/**Supplementary Material**, further inquiries can be directed to the corresponding author.

## Author Contributions

JY, QZ, and XW conceived the idea and contributed to the study design, discussed the results, and wrote the manuscript. JY, WS, and XZ performed data collection in the field and contributed to chronology data analysis. WS and XZ performed meteorological data collection and analysis. XW funded the study. All authors contributed to interpreting the results, discussion, and approved the final manuscript.

## Funding

This work was supported by the Key Project of the China National Key Research and Development Program (2021YFD2200401), the National Natural Science Foundation of China (41877426), and the Fund of Eco-meteorological Innovation Open Laboratory in Northeast China, China Meteorological Bureau (stqx2018zd02).

## Conflict of Interest

The authors declare that the research was conducted in the absence of any commercial or financial relationships that could be construed as a potential conflict of interest.

## Publisher’s Note

All claims expressed in this article are solely those of the authors and do not necessarily represent those of their affiliated organizations, or those of the publisher, the editors and the reviewers. Any product that may be evaluated in this article, or claim that may be made by its manufacturer, is not guaranteed or endorsed by the publisher.

## Footnotes

^1^ http://climexp.knmi.nl
