# Supplementary material for: Radial Growth of Trees Rather Than Shrubs in Boreal Forests Is Inhibited by Drought
Source: Front Plant Sci. 2022 Jun 2;13:912916. doi: 10.3389/fpls.2022.912916 (PMC9201406; doi:10.3389/fpls.2022.912916)
Supplement: Supplementary file 1 [file Data_Sheet_1.PDF]

Supplementary Material

Table S1 Summary statistics of all chronologies in this study.

Code	Age	SD	AC1	MS	R_{bar}	EPS	SNR	VF1 (%)
XLLG	159	0.32	0.64	0.23	0.43	0.96	23.3	46.66
YKLG	285	0.33	0.77	0.21	0.30	0.88	7.60	29.40
AGLLG	239	0.20	0.50	0.16	0.34	0.96	21.08	37.01
ALSLG	116	0.22	0.43	0.20	0.32	0.91	9.59	37.23
MELG	320	0.28	0.69	0.18	0.29	0.85	5.40	31.80
QQLG	270	0.27	0.6	0.19	0.24	0.89	8.37	29.02
YKPS	315	0.29	0.47	0.19	0.35	0.95	18.40	48.70
YAPS	313	0.27	0.73	0.15	0.41	0.90	12.30	39.20
QQPS	287	0.24	0.72	0.15	0.29	0.95	18.22	32.10
FHPS	138	0.23	0.75	0.13	0.34	0.95	20.77	37.10
FKPS	168	0.25	0.58	0.19	0.39	0.95	18.18	42.81
MHPS	187	0.30	0.72	0.19	0.32	0.93	13.29	35.75
SLPS	145	0.31	0.84	0.15	0.52	0.97	28.43	54.72
ALPS	154	0.20	0.52	0.17	0.25	0.88	7.07	31.59
LHPP	81	0.14	0.84	0.14	0.18	0.89	8.09	27.22
LLPP	68	0.21	0.70	0.13	0.27	0.93	13.00	37.09
TSPP	111	0.22	0.80	0.12	0.1	0.85	3.90	19.61
XBPP	69	0.16	0.38	0.14	0.21	0.92	12.15	24.5
HMPP	127	0.21	0.46	0.17	0.22	0.90	14.81	26.98
ZLPP	69	0.23	0.81	0.19	0.24	0.92	10.75	29.49
FKPP	134	0.23	0.55	0.17	0.19	0.87	6.66	24.76
ALPP	72	0.32	0.81	0.15	0.27	0.89	8.27	37.92
AHPP	108	0.36	0.68	0.15	0.14	0.85	3.63	27.74
DLPP	79	0.29	0.80	0.12	0.21	0.91	10.30	37.1
DMPP	70	0.19	0.56	0.14	0.16	0.85	4.42	26.82
DHPP	89	0.21	0.61	0.15	0.21	0.87	6.76	26.87

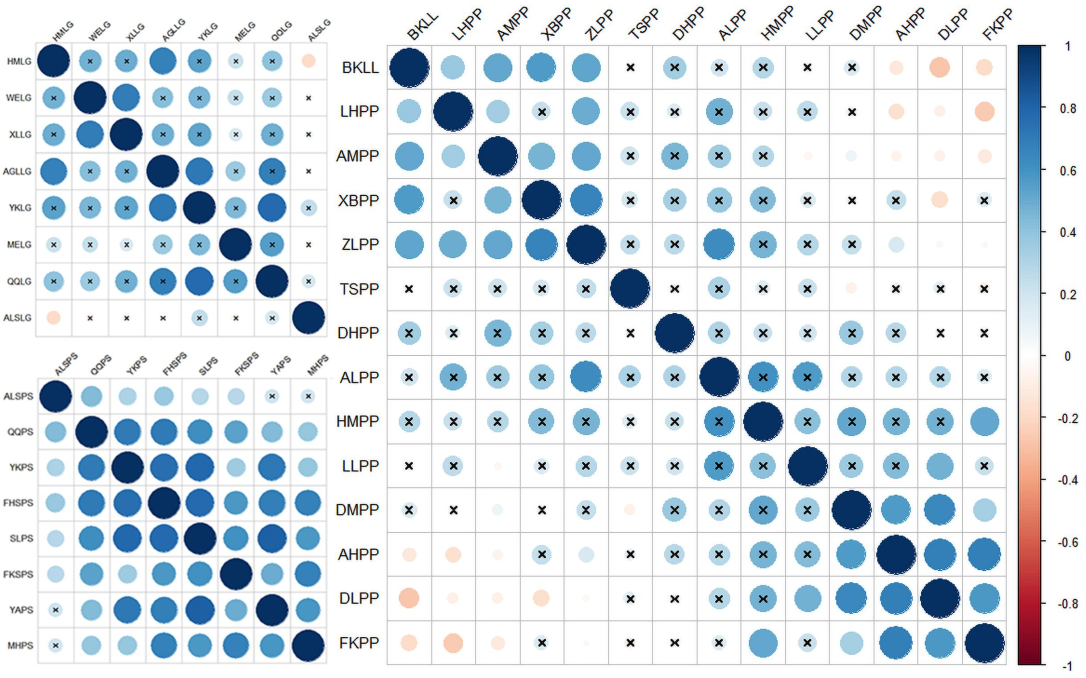

Figure S1. Pearson correlations calculated among *Pinus pumila*, *Pinus sylvestris* var. *mongolica* and *Larix gmelinii* chronologies (1950-2014). The blue circle represents a positive correlation while red is a negative correlation. The size of the circle represents the strength of the correlation. The”x”represents p>0.05.

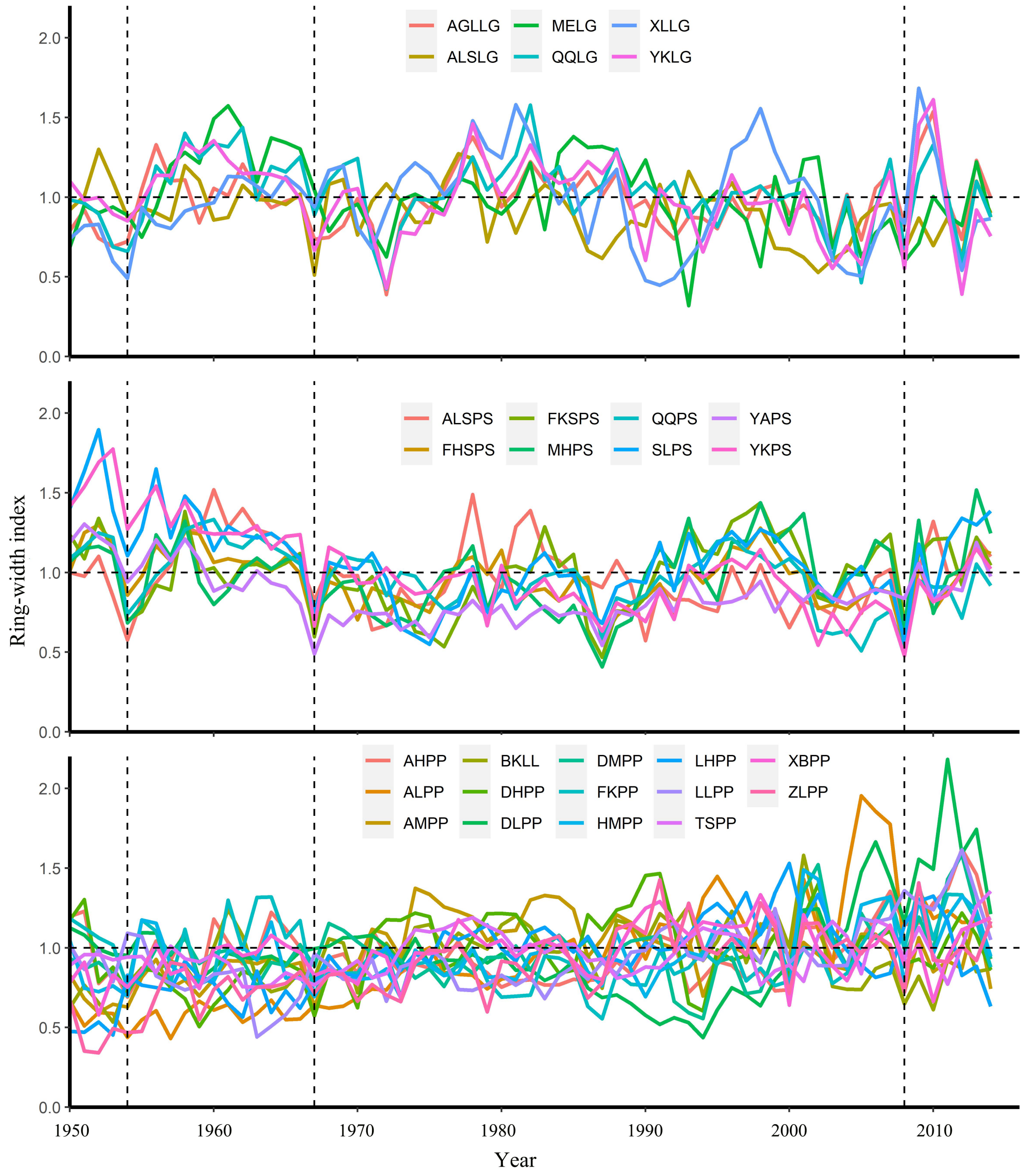

Fig. S2. Changes in the ring-width index of *L. gmelinii*, *P. pumila* and *P. sylvestris* var. *mongolica* in northeast China, the vertical dash lines represent drought years(1954, 1967, 2008).

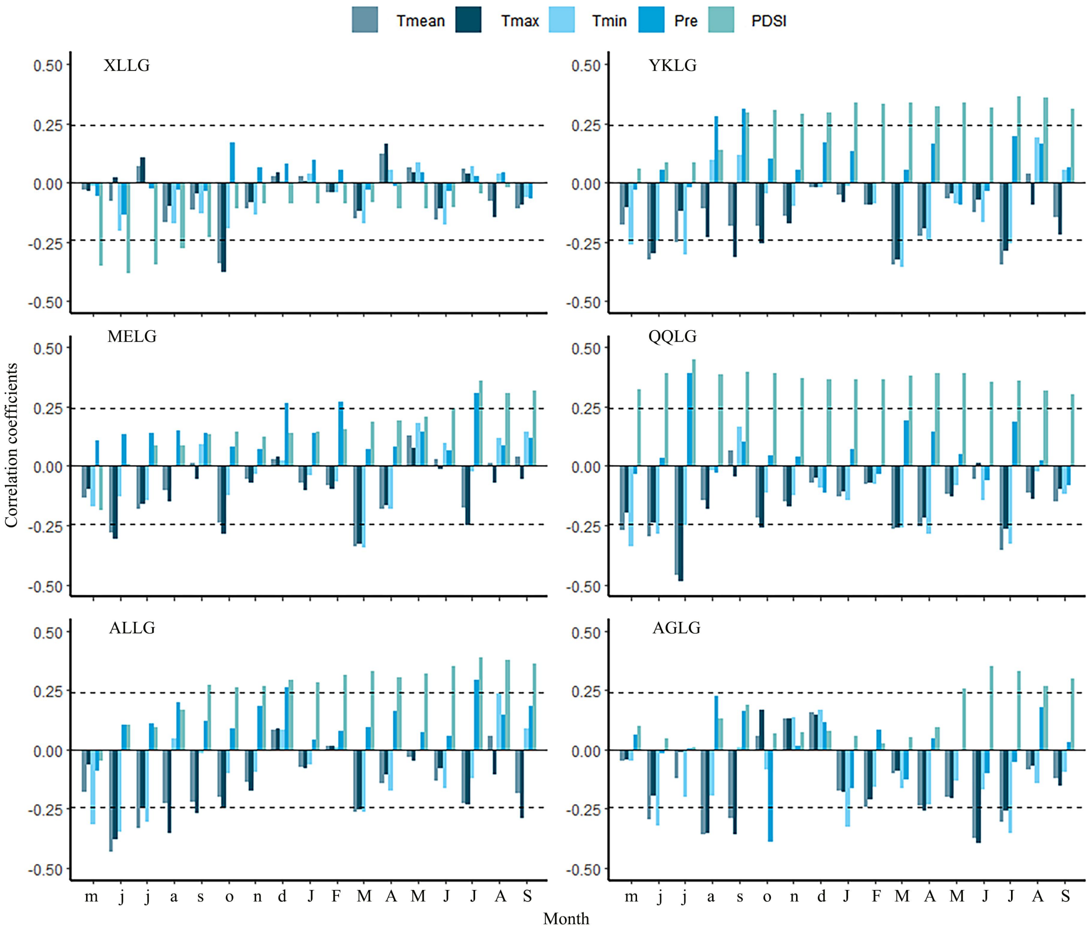

Fig. S3. Pearson correlation of *L. gmelinii* ring-width index with monthly climate data from previous May (lower case m) to current September (upper case S) during 1950–2014. Horizontal dashed lines indicate the 95% significance levels.

Notes: Tmean-monthly mean temperature, Tmax-monthly maximum temperature, Tmin-monthly minimum temperature, Pre-monthly precipitation, PDSI-Palmer Drought Severity Index.

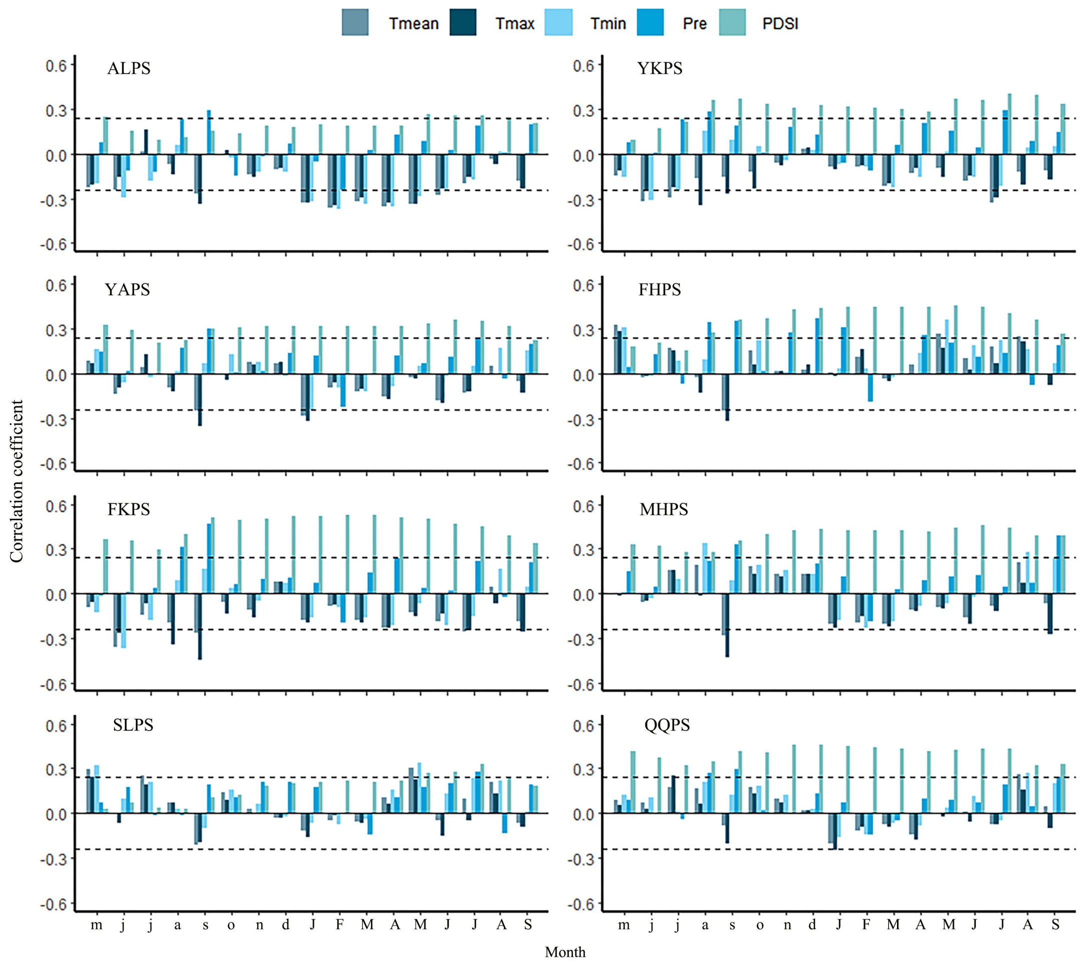

Fig. S4. Pearson correlation of *P. sylvestris* var. *mongolica* ring-width index with monthly climate data from previous May (lower case m) to current September (upper case S) during 1950–2014. Horizontal dashed lines indicate the 95% significance levels.

Notes: Tmean-monthly mean temperature, Tmax-monthly maximum temperature, Tmin-monthly minimum temperature, Pre-monthly precipitation, PDSI-Palmer Drought Severity Index.

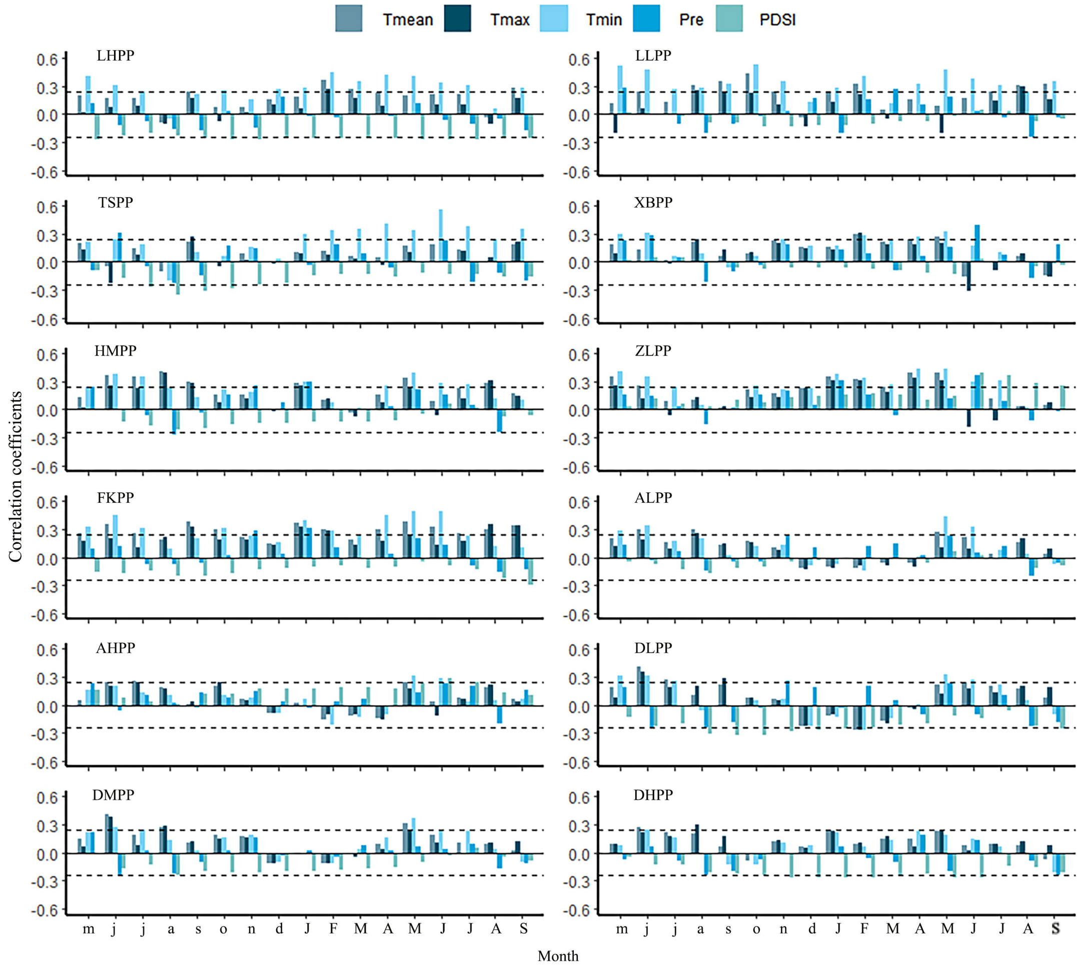

Fig. S5. Pearson correlation of *P. pumila* ring-width index with monthly climate data from previous May (lower case m) to current September (upper case S) during 1950–2014. Horizontal dashed lines indicate the 95% significance levels.

Notes: Tmean-monthly mean temperature, Tmax-monthly maximum temperature, Tmin-monthly minimum temperature, Pre-monthly precipitation, PDSI-Palmer Drought Severity Index.

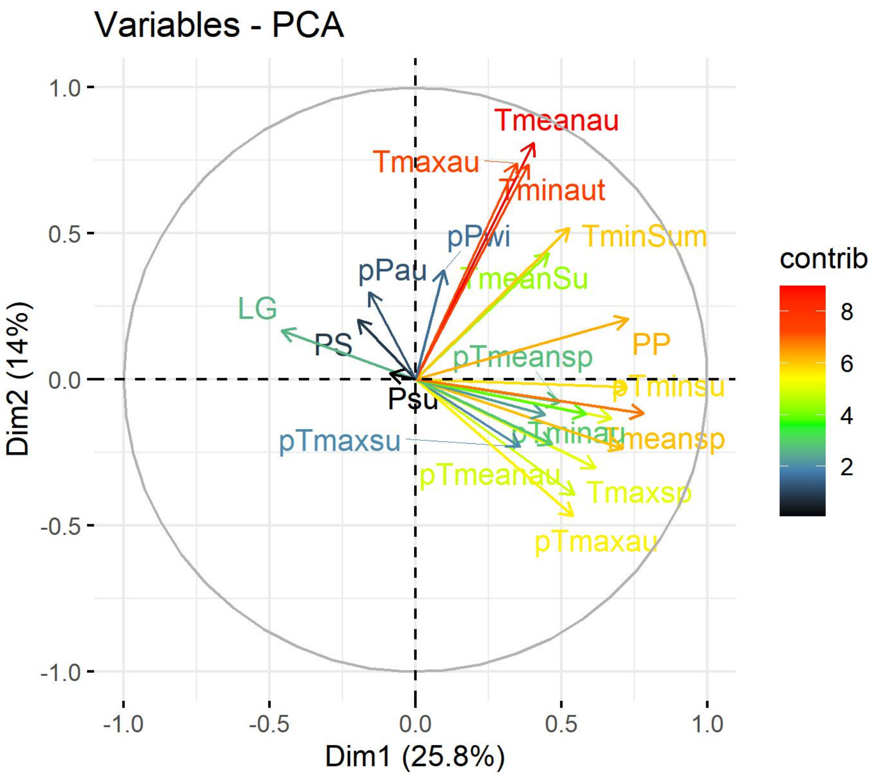

Fig. S6 Principal component analysis based on the three species ring-width indices and seasonal climatic factors.

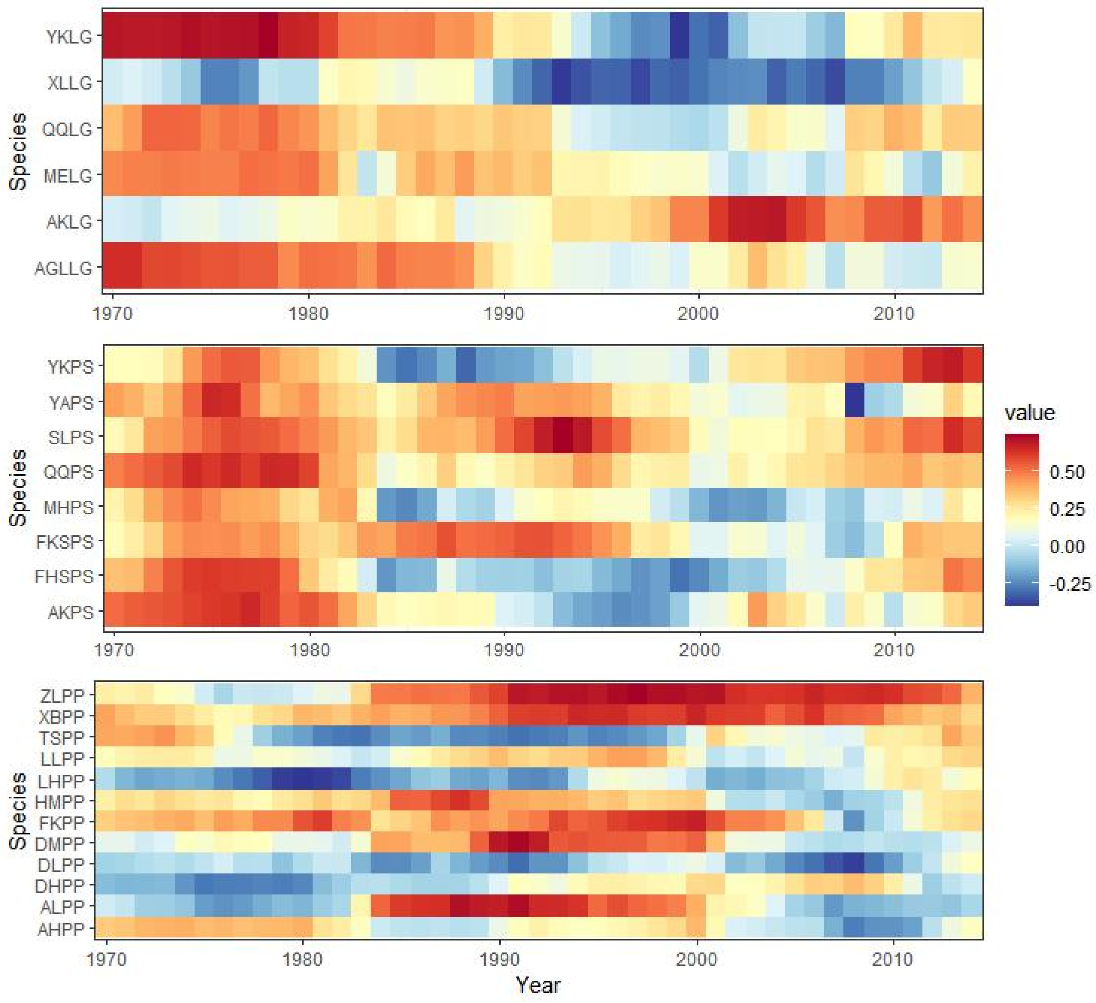

Fig. S7. The 21-year moving correlation analysis between the ring-width index of three species and minimum PDSI from June to August during the period 1970-2014.

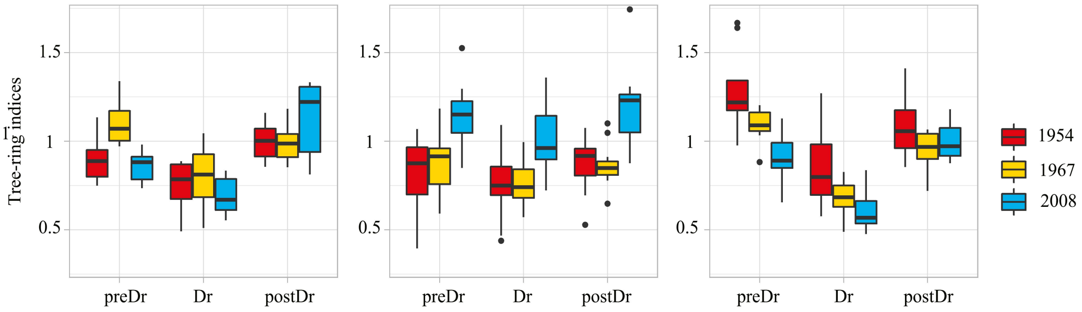

Figure. S8. Boxplot of three species ring-width index before, during and after drought years.

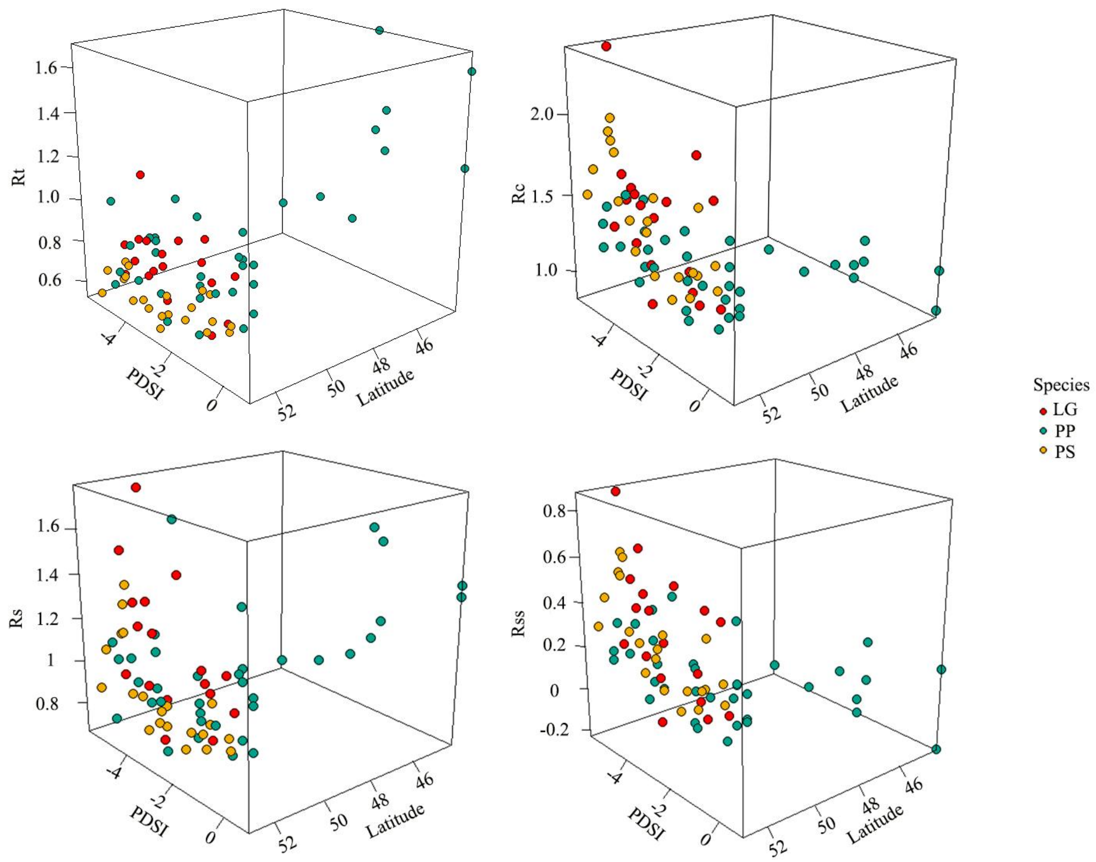

Fig. S9. Scatterplots of regional *L. gmelinii*, *P. pumila* and *P. sylvestris* var. *mongolica* resistance, recovery, resilience and the relative resilience with PDSI and latitude.
